# Translated Emission Pathways (TEPs): Long‐Term Simulations of COVID‐19 CO_2_ Emissions and Thermosteric Sea Level Rise Projections

**DOI:** 10.1029/2021EF002453

**Published:** 2022-08-24

**Authors:** Alan R. Gonzalez, Ting Lin

**Affiliations:** ^1^ Department of Civil Environmental, and Construction Engineering Texas Tech University Lubbock TX USA

**Keywords:** emissions, COVID‐19, sea level rise, climate change, radiative forcing, science communication

## Abstract

Within the scientific community, climate models have been established to relate long‐term emission scenarios to their respective environmental response. Although data at high resolutions can be obtained, this research framework is often computationally complex and offers limited readability for the general public. With the COVID‐19 pandemic bringing forth a new sense of lifestyle and reduced human activity, the CO_2_ emission data related to this global event can be used to illustrate the context of climate science to a broader audience. This study proposes a series of translated emission pathways (TEPs) that consist of CO_2_ emission patterns from the various phases of COVID‐19 response and demonstrate a resemblance to the forcing scenarios utilized within climate research. A simple climate model and radiative forcing expression are used to parameterize the CO_2_ emission data from the TEPs to its respective atmospheric conditions. Thermosteric sea level rise is used as a metric of environmental impact to highlight the differences between the TEPs. By referencing the COVID‐19 pandemic and establishing a linear research framework, this study introduces climate research to the general public and serves as a call to action for environmental responsibility.

## Key Points

- The CO_2_ emissions of the COVID‐19 pandemic can be used to illustrate climate research to a broader audience
- Long‐term simulations of COVID‐19 CO_2_ emissions convey feasibility for climate change mitigation
- Thermosteric sea level rise is an effective form of data output that can clearly demonstrate the environmental effects of human activity

## 1 Introduction

Due to the imminent effects it can pose on the world and its inhabitants, climate change has become one of the most notable concerns of the 21st century. As an interdisciplinary topic, reliable projections of environmental data and effective forms of science communication are necessary to address this serious issue. Although many scientific advancements have been made to recognize the sources and impacts of climate change, mitigation strategies are frequently questioned by the general public due to varying levels of interest on the matter and difficulty in understanding scientific data (Leiserowitz et al., 2021). In order to form consensus on the climate change recommendations made by the scientific community, it is vital to develop actionable science that illustrates climate research to the general public while emphasizing the importance of their environmental consideration. Simulating the influence of human activity on climate change—in the context of the COVID‐19 pandemic—can help to achieve these goals.

The COVID‐19 pandemic has recently instilled communities around the world with a different sense of lifestyle and normality. With an intent to limit the repercussions of the COVID‐19 pandemic, different magnitudes of guidelines and regulations were implemented during initial lockdown periods and subsequent reopening phases. Among some of the factors related to COVID‐19 response were a decrease in social gatherings, travel restrictions, and limits on business operation. Due to this form of reduced human activity, instantaneous changes in environmental conditions were recognized around the world (Fu et al., 2020; Le et al., 2020; Le Quéré et al., 2020; Turner et al., 2020; Yadav et al., 2021; Yang et al., 2020). As described by Liu et al. (2020), the decrease in CO_2_ emissions during the first 6 months of 2020, when compared to the same period in 2019, was larger than that of recent economic downturns or World War II. Although this decrease in CO_2_ emissions is an instantaneous change when compared to the steady increases that have occurred since the 1940s, the COVID‐19 pandemic provides a preview of the environmental benefits that can be obtained if climate change mitigation becomes a sustained practice.

Although COVID‐19 circumstances have previously been related to climate forcing scenarios (Lamboll et al., 2021), this study focuses on utilizing global CO_2_ emission patterns—during different phases of the COVID‐19 pandemic—to develop a series of long‐term pathways that represent various magnitudes of climate change mitigation. A simple climate model and radiative forcing expression are used to relate the CO_2_ emission data to its respective atmospheric conditions. Often recognized as one of the most critical impacts of climate change, sea level rise projections are incorporated into this study to further emphasize the positive environmental effects of mitigation efforts. While considering readability for the general public, forming connections between climate research and the tangible experiences of the COVID‐19 pandemic can promote widespread scientific literacy.

## 2 Materials and Methods

**Figure 1:**
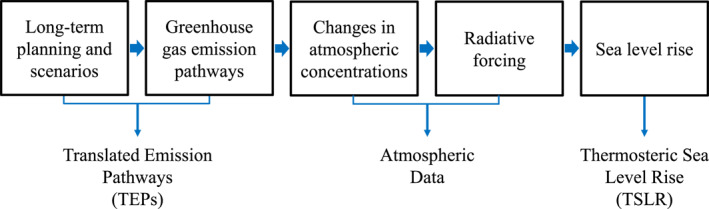
Research framework used in this study to relate COVID‐19 CO_2_ emissions to future thermosteric sea level rise (TSLR).

To provide coherence for a broader audience, this research study developed the COVID‐19 CO_2_ emission pathways and their respective sea level rise projections through a linear procedure. Figure 1 depicts the methodology that directed this study.

In order to facilitate the evaluation of future climate change, a variety of social, economic, and environmental factors have been formulated into long‐term, forcing scenarios. Among these established scenarios are those described through representative concentration pathways (RCPs) (Meinshausen et al., 2011; Moss et al., 2010). Based on varying levels of long‐term activity and mitigation emphasis, RCPs provide projections for the emissions and atmospheric concentrations of various greenhouse gases and aerosols. Due to its significant contribution to total anthropogenic emissions, CO_2_ emissions are commonly regarded as a strong indicator of climate conditions. Furthermore, CO_2_ equivalent (CO_2_‐eq) emissions and concentrations are often used as metrics in climate research to aggregate the effects of other greenhouse gases (e.g., CH_4_ and N_2_O). Resulting from the novelty of COVID‐19, however, many statistical conclusions regarding the emissions and consequential atmospheric concentrations of all greenhouse gases have yet to become abundant throughout literature. Despite these limitations on immediate data, various methods for the real‐time monitoring of CO_2_ emissions have been constructed. Through a sectoral analysis, the Carbon Monitor initiative (Liu et al., 2020) provides regional and global approximations of CO_2_ emissions during the COVID‐19 pandemic. While incorporating various design parameters to resemble the RCPs, COVID‐19 CO_2_ emission patterns were formulated into a series of translated emission pathways (TEPs) that illustrate climate research in the context of the ongoing pandemic.

To prepare for a sea level rise analysis, the CO_2_ emissions of the TEPs were related to their respective CO_2_ atmospheric concentrations. Tracing the influence of instantaneous CO_2_ emissions on future CO_2_ atmospheric concentrations requires an intricate evaluation of the carbon cycle and climate system as a whole. This study utilized the Bern Simple Climate Model (BernSCM) which consists of a series of equations that budget carbon and heat fluxes between the atmosphere, ocean, and land biosphere (Strassmann & Joos, 2018). A forcing file containing the CO_2_ emissions from the TEPs and supplementary non‐CO_2_ radiative forcing from the RCP Database (Meinshausen et al., 2011) was supplied to the BernSCM to estimate the TEP CO_2_ concentrations. Simplified methods for obtaining sea level rise projections (e.g., Rahmstorf, 2007; Vermeer & Rahmstorf, 2009) use radiative forcing, or the net energy change of the Earth's climate system, as an independent variable. To obtain these values, the CO_2_ atmospheric concentrations from the TEPs were applied to the equation in Table 3 of Myhre et al. (1998) that outputs radiative forcing due to CO_2_. This radiative forcing data set provided the primary variable used in the TSLR analysis.

Although ongoing research aims to reduce the uncertainty of sea level rise projections, including the influence of glacier melt (e.g., Radić et al., 2013) and ice sheet conditions (e.g., Nowicki et al., 2013), thermal ocean expansion is often considered the most significant contributor to future sea level rise and a direct measure of climate change conditions. This study determined the thermosteric sea level rise (TSLR) caused by the TEPs to further emphasize their environmental consequences. With reference to Thomas and Lin (2018), the nonlinear response functions expressed in Equations 2–4 and the parameters for the NorESM1‐m model were used to relate the CO_2_ radiative forcing data set of the TEPs to their projected TSLR. This form of data output provides an example of how individual actions, as suggested by COVID‐19 response, can affect the magnitude of the damaging repercussions caused by climate change.

## 3 Data

**Figure 2:**
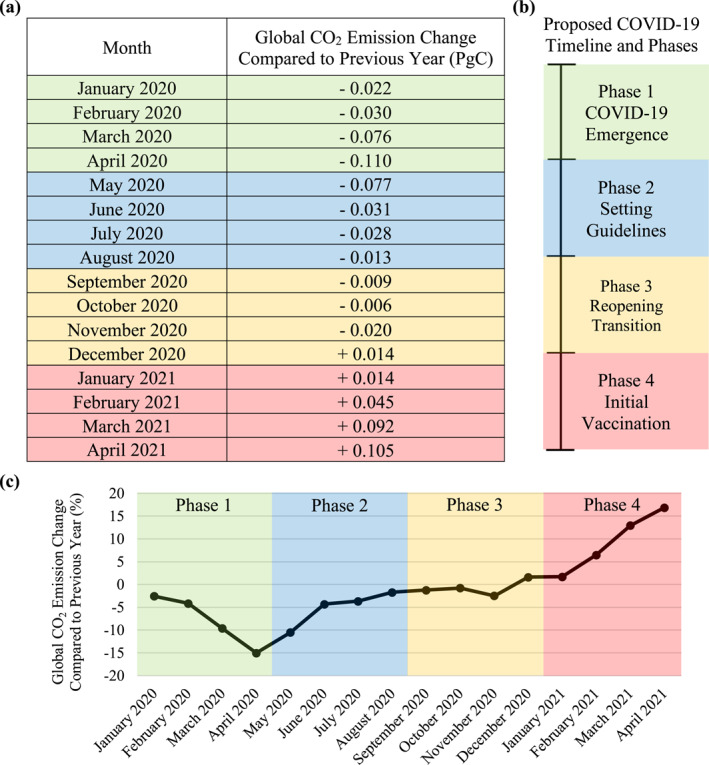
Changes in global CO_2_ emissions throughout 2020 and 2021 based on values provided by the carbon monitor initiative (Liu et al., 2020). (a) Monthly global CO_2_ emissions were compared to their respective values from the previous year. (b) While utilizing the timeline of WHO's response to COVID‐19 as an international reference (World Health Organization, 2022), the proposed COVID‐19 timeline divides the 16‐month study period from January 2020—April 2021 into four phases. Phase 1 describes the initial emergence of the COVID‐19 pandemic. With over 1 million reported COVID‐19 cases, Phase 2 represents the continued implementation of national guidelines and regulations to prevent further spread of the disease. Phase 3 consists of the development of equitable vaccine access plans and the release of guidance on risk‐based international travel. Phase 4 comprises of initial vaccination rollouts with COVAX delivering more than 38 million doses. (c) A visual representation of yearly changes in global CO_2_ emissions as percentages conveys the distinct characteristics of each COVID‐19 phase.

Highly dependent on national conditions, such as population density and economic flexibility, countries around the world instituted different regulations to control the spread of COVID‐19 during lockdown periods and subsequent reopening phases. While also considering the emergence of COVID‐19 variants, general assumptions would be needed to define COVID‐19 phases that are representative of global circumstances. However, a COVID‐19 timeline has been recorded by the World Health Organization (WHO) that documents the release of international guidance and plans for promoting equitable vaccine access, amongst other resources (World Health Organization, 2022). Based on these dates and as further described in Figure 2, the 16‐month period considered in this study divided the events of the COVID‐19 pandemic into four phases. Global CO_2_ emissions for each month within this study period were obtained from the Carbon Monitor initiative (Liu et al., 2020) and compared to respective values from the previous year. This method of data analysis was selected with a consideration to the natural and seasonal changes in CO_2_ emissions that can appear throughout the year and to maintain a uniformly spaced point of reference. Figure 2 details the yearly changes in global CO_2_ emissions during each month of the proposed COVID‐19 timeline.

## 4 Results and Discussion

### 4.1 Translated Emission Pathways (TEPs)

In order to resemble the four common RCPs (Meinshausen et al., 2011; Moss et al., 2010), four TEPs were constructed based on the global CO_2_ emission changes noted throughout the COVID‐19 pandemic. Historical CO_2_ emissions used to construct the TEPs were obtained from the Global Carbon Project (Friedlingstein et al., 2020). Since historical CO_2_ emission data was considered until the year 2020, 80 years of simulated data were needed to complete a series of 100‐year projections. Although conditions and criteria vary among the TEPs, each scenario utilized the COVID‐19 CO_2_ emission change data, as presented in Figure 2, and translated these values across several years. In this case, the rather quick changes in CO_2_ emissions that occurred during the COVID‐19 pandemic were simulated in a longer‐term setting to account for a more intentional emission pathway.

Throughout the TEPs, single or combined phases of COVID‐19 CO_2_ emissions were selected to resemble the various emission pathways and mitigation emphasis of the RCPs. In addition, a monthly multiplier was utilized to scale the different pathways to match the RCP projections more closely. This multiplier considers the number of months in each year that must meet the emission requirements in order to maintain the proposed TEP, all other months compensating for each other or demonstrating no change in emissions. Furthermore, the order of emission data was adjusted to promote a more natural transition between values. Table 1 summarizes the conditions used to develop each TEP and is followed by detailed explanations.

**Table 1:** Conditions Used to Construct the Translated Emission Pathways (TEPs)

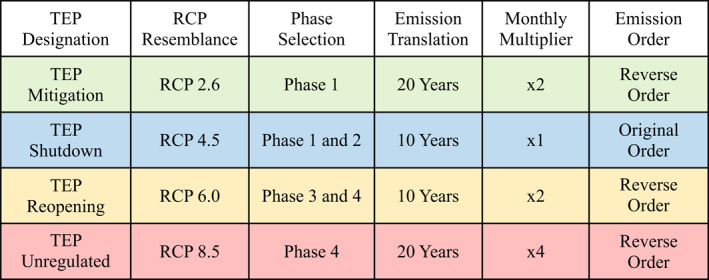

*Note*. In order to resemble its respective RCP, each TEP followed a similar CO_2_ emission pattern as suggested by the various phases of COVID‐19 response. To complete a 100‐year period of projections, changes in CO_2_ emissions, as shown in Figure 2, were translated across multiple years. A monthly multiplier was incorporated to represent the number of months in each year that must meet the emission standards in order to satisfy the TEP conditions, all other months demonstrating no change in emissions or compensating for each other. The emission order was used to adjust CO_2_ phase emissions so that they followed a more seamless transition.

TEP Mitigation—Resembles RCP 2.6 and was constructed from the CO_2_ emission trends from Phase 1 of the COVID‐19 timeline. CO_2_ emission values were translated across 20 years in order to complete the pathway under a 100‐year period. TEP Mitigation utilized a x2 multiplier to demonstrate similar emissions to RCP 2.6. Emission values from Phase 1 were presented in reverse order to replicate larger decreases in CO_2_ emissions during the earlier years of the simulation period.

TEP Shutdown—Resembles RCP 4.5 and consists of CO_2_ emission values from Phase 1 and Phase 2 of the COVID‐19 timeline. Each emission value from both phases was translated across 10 years and is composed of a x1 multiplier. The original order of emission values from Phase 1 and Phase 2 was maintained.

TEP Reopening—Resembles RCP 6.0 and consists of CO_2_ emission values from Phase 3 and Phase 4 of the COVID‐19 timeline. The emission changes from each phase were translated across 10 years and make use of a x2 multiplier. TEP Reopening presents emission values in reverse order to replicate an initial increase in CO_2_ emissions similar to RCP 6.0.

TEP Unregulated—Resembles RCP 8.5 and utilized CO_2_ emission values from Phase 4 of the COVID‐19 timeline. Similar to TEP Mitigation, each emission value was translated across 20 years to complete the pathway. A multiplier of x4 was used to scale the emission trends. The changes in CO_2_ emissions were used in reverse order to demonstrate larger emission increments toward the earlier years of the modeling period.

**Figure 3:**
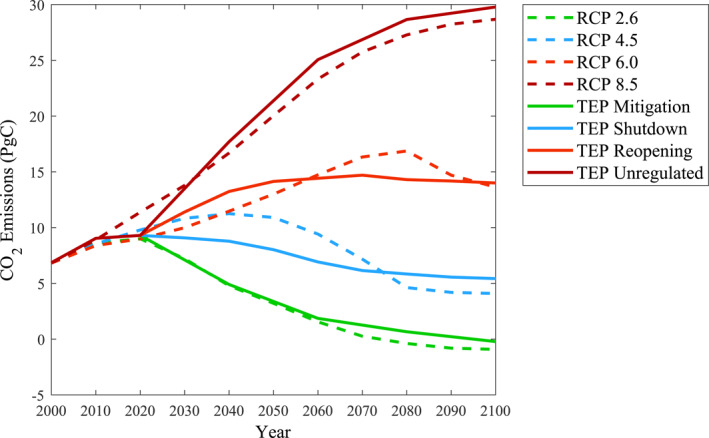
Global CO_2_ emissions of the translated emission pathways (TEPs) and representative concentration pathways (RCPs) (Meinshausen et al., 2011; Moss et al., 2010) under a 100‐year period. Historical data used to construct the TEPs was obtained from the Global Carbon Project (Friedlingstein et al., 2020) until the year 2020. In order to satisfy the 80 years of future projections, each TEP translated CO_2_ emission changes from the various phases of the COVID‐19 pandemic across multiple years. Additional factors used to construct the various TEPs are shown in Table 1.

By establishing the TEP conditions, CO_2_ emission trendlines based on COVID‐19 response were used to replicate the RCPs. Figure 3 depicts the TEP CO_2_ emissions plotted alongside the RCPs for comparison.

As shown in Figure 3, similarities are shared between the TEPs and RCPs. By referencing the different phases of the COVID‐19 pandemic, the TEPs support the scientific context of the RCPs in a format that can be understood by the general public. It is also important to note the differences in CO_2_ emissions seen throughout the study period but ultimate similitude between the TEPs and RCPs in the year 2100. This detail highlights how long‐term CO_2_ emission goals can be met through steady emission pathways, regardless of intermediate differences between the TEP and RCP projections. Many times, conversation on whether it is too late to mitigate the effects of climate change is discussed amongst the general public. Maintaining TEP CO_2_ emission trends, as seen through the phases of COVID‐19 response, reflects both feasibility and flexibility for meeting long‐term RCP criteria.

The COVID‐19 phases used to construct each TEP provide a more tangible demonstration of the CO_2_ emission pathways used within climate research. Selecting Phase 1 and Phase 4 of the COVID‐19 pandemic as the dictating criteria for TEP Mitigation and TEP Unregulated, respectively, effectively contrasts the environmental response associated to polar levels of climate change mitigation. Meanwhile, TEP Shutdown and TEP Reopening serve as moderate pathways that represent smaller changes in present‐day CO_2_ emissions. In addition, the monthly multipliers are vital for demonstrating the feasibility of the various TEPs. This single parameter conveys to the general public how meeting long‐term RCP conditions does not require exact replications of emission changes from every month of the COVID‐19 study period. Contrary, counteracting positive and negative emission changes throughout the majority of months in each year is recognized for its ability to accomplish desirable CO_2_ emissions. If long‐term emission goals are to be met, it is vital to encourage net‐zero mitigation strategies and research that pursues negative emissions, where more CO_2_ is removed from the atmosphere than what is released.

**Figure 4:**
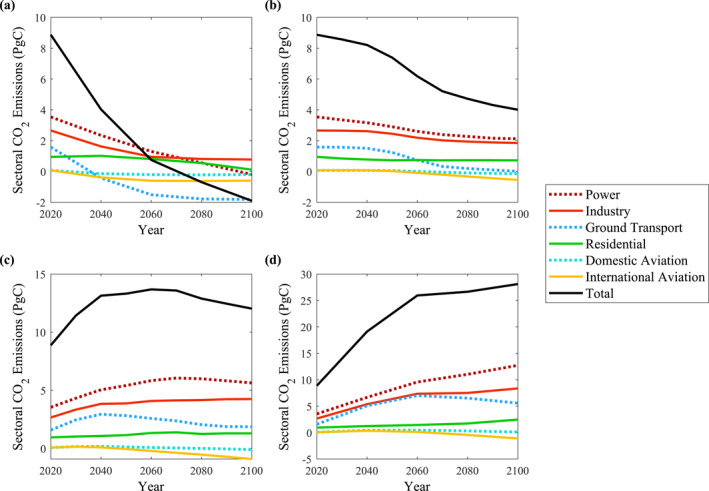
Global sectoral CO_2_ emissions provided by the Carbon Monitor initiative (Liu et al., 2020) were subjected to the criteria of (a) translated emission pathway (TEP) Mitigation, (b) TEP Shutdown, (c) TEP Reopening, and (d) TEP Unregulated in order to highlight the impact of individual decision making on achieving long‐term climate goals.

Similar to the initial construction of the TEPs, performing a sectoral analysis of CO_2_ emissions further conveys the relationship between individual decision making and meeting long‐term climate goals. Utilizing the global sectoral CO_2_ emissions provided by the Carbon Monitor initiative (Liu et al., 2020), monthly emission averages were determined for the COVID‐19 timeline under the sectors of power, industry, ground transport, residential, domestic aviation, and international aviation. The TEP criteria, as shown in Table 1, were then utilized to provide long‐term simulations of CO_2_ emission data. Figure 4 depicts the global sectoral CO_2_ emissions until the year 2100 based on the TEP conditions.

As shown throughout Figure 4, TEPs that included a decrease in CO_2_ emissions among the sectors of power, industry, and ground transport were attributed to significantly lower long‐term emissions. In addition to the potential systematic changes that could contribute to reduced CO_2_ emissions in these sectors, this detail emphasizes the importance of individual decision making and the necessity for overall environmental consensus. Whether it be at home, school, or work, this study encourages individuals to become aware of their influence on total CO_2_ emissions and re‐evaluate their everyday decisions. As suggested through the Intergovernmental Panel on Climate Change (IPCC) and the recent Mitigation of Climate Change part of its Sixth Assessment Report (AR6), long‐term climate goals can be met if all members of society are able to collectively practice and implement environmentally considerate choices, such as reducing energy consumption and incorporating sustainable forms of transportation into daily commutes (IPCC, 2022a).

Being able to create a clear parallel between the human activity related to each phase of the COVID‐19 pandemic and its associated environmental response provides the general public with an understanding of what the RCPs entail. Having recognized the lifestyles and emission trends of the COVID‐19 phases, feasibility for mitigation pathways and the actions necessary to meet scenario requirements are promoted. Furthermore, satisfying long‐term climate goals would only require maintaining the emission patterns described through the TEPs. This form of science communication effectively conveys climate change data to the general public while developing consensus and encouraging support for mitigations strategies.

### 4.2 Atmospheric Data

**Figure 5:**
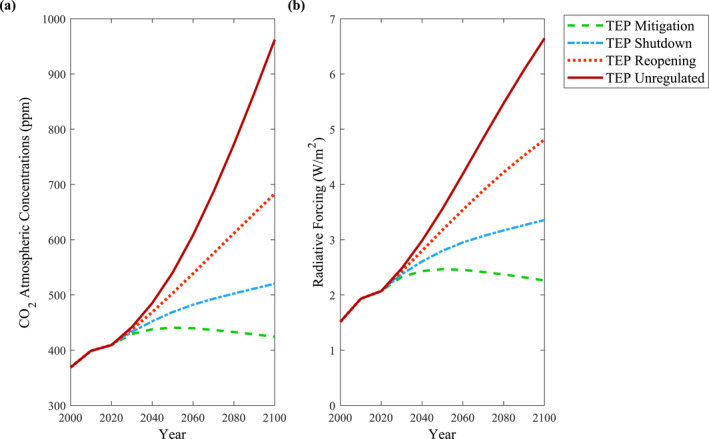
Based on the translated emission pathway (TEP) CO_2_ emissions, (a) CO_2_ atmospheric concentrations were obtained by implementing the Bern Simple Climate Model (Strassmann & Joos, 2018) and (b) CO_2_ radiative forcing values were calculated using the representative equation established by Myhre et al. (1998).

The BernSCM (Strassmann & Joos, 2018) was used to relate the CO_2_ emissions of the various TEPs to their respective CO_2_ atmospheric concentrations. Historical CO_2_ atmospheric concentrations and non‐CO_2_ radiative forcing values were referenced from the RCP Database (Meinshausen et al., 2011). Figure 5a demonstrates the CO_2_ atmospheric concentrations of the TEPs. Using the simplified expression established by Myhre et al. (1998) and as shown in Figure 5b, CO_2_ radiative forcing data was determined based on the CO_2_ atmospheric concentrations of the TEPs.

Similar to the CO_2_ emission trends shown in Figure 3, the atmospheric response of the various TEPs resonate the importance of mitigation and wise decision making. Recalling the human activity and personal experiences of the COVID‐19 pandemic can aid the general public in understanding what each emission pathway constitutes and their related environmental impact. In addition, the CO_2_ radiative forcing data from the TEPs served as the primary variable for the TSLR analysis that followed.

### 4.3 Thermosteric Sea Level Rise (TSLR)

**Figure 6:**
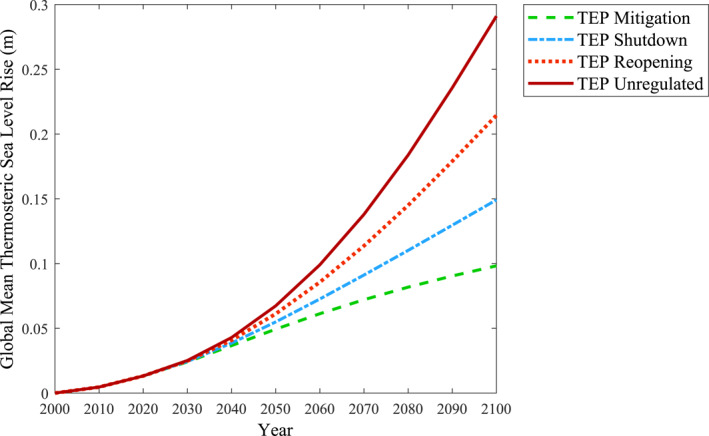
Thermosteric sea level rise output is obtained by utilizing the CO_2_ radiative forcing values of the translated emission pathways (TEPs) and implementing the nonlinear response functions under the NorESM1‐m model as proposed by Thomas and Lin (2018).

To further communicate the environmental responses associated to the TEPs, TSLR based on the influence of CO_2_ was selected as a clear and effective form of data output. The nonlinear response functions and parameters for the NorESM1‐m model established by Thomas and Lin (2018) were used to relate the CO_2_ radiative forcing values of the TEPs to their respective TSLR. Figure 6 demonstrates the TSLR associated to the forcing conditions of the various TEPs.

As depicted in Figure 6 and when compared to TEP Unregulated, pursuing the emission trends of TEP Mitigation can lead to an approximate TSLR decrease of 0.2 m by the year 2100. Considering the endangerment of communities and ecosystems that could result from higher magnitudes of TSLR, it is important to convey to the public how this form of climate response can be avoided if lower CO_2_ emission pathways are followed. When considering the conditions set forth by the TEPs, pursuing emission trends reminiscent to those seen during Phase 1 and Phase 2 of the COVID‐19 timeline can promote a more favorable TSLR response. By performing this TSLR analysis, the environmental implications of the TEPs and the importance of climate change mitigation are further conveyed to the general public.

## 5 Conclusion

In order to lessen the impact of climate change, it is clear that a steady decrease in anthropogenic CO_2_ emissions is necessary. Based on the similarities between COVID‐19 CO_2_ emission patterns and the long‐term scenarios used in climate research, this study provides a preview of what the future might look like if widespread environmental consensus is established. Developing the TEPs allows the general public to better perceive the scientific and social context of low‐emission scenarios. Furthermore, the research framework implemented into this study and the resulting TSLR projections provide a clear illustration of the environmental benefits related to a long‐term reduction of CO_2_ emissions. In this manner, strategies recommended by the scientific community to cope with the effects of climate change can be received with a sense of understanding and support. Since climate change will affect all walks of life around the world, this form of science communication and social action are of utmost importance. As the Intergovernmental Panel on Climate Change (IPCC, 2022b) is releasing its Sixth Assessment Report (AR6) and the United Nations Framework on Climate Change (UNFCCC, 2022) responds to recent COP26 discussion, this research offers a promising start to communicate climate science—by translating COVID‐19 CO_2_ emissions to future sea level rise projections—In order to inspire collective action amid the pandemic.

## Supporting information

Data Set S1

## Data Availability

Liu et al. (2020). The Carbon Monitor initiative provides public access to regularly updated estimates of sectoral CO_2_ emissions. https://doi.org/10.1038/s41467-020-18922-7. Strassmann and Joos (2018). The Bern Simple Climate Model (BernSCM) is an open‐source climate model that utilizes carbon cycle analysis and parameterization to estimate climate system response. https://doi.org/10.5194/gmd-11-1887-2018. Datasets and code used to construct the proposed TEPs and their environmental response, as shown in the figures of this study, are publicly accessible through the Zenodo repository at https://doi.org/10.5281/zenodo.6506929
