# Supplementary material for: Translated Emission Pathways (TEPs): Long‐Term Simulations of COVID‐19 CO2 Emissions and Thermosteric Sea Level Rise Projections
Source: Earths Future. 2022 Aug 24;10(8):e2021EF002453. doi: 10.1029/2021EF002453 (PMC9538853; doi:10.1029/2021EF002453)

(a)

Month	Global CO_2 Emission Change Compared to Previous Year (PgC)
January 2020	- 0.022
February 2020	- 0.030
March 2020	- 0.076
April 2020	- 0.110
May 2020	- 0.077
June 2020	- 0.031
July 2020	- 0.028
August 2020	- 0.013
September 2020	- 0.009
October 2020	- 0.006
November 2020	- 0.020
December 2020	+ 0.014
January 2021	+ 0.014
February 2021	+ 0.045
March 2021	+ 0.092
April 2021	+ 0.105

(b)

Proposed COVID-19 Timeline and Phases

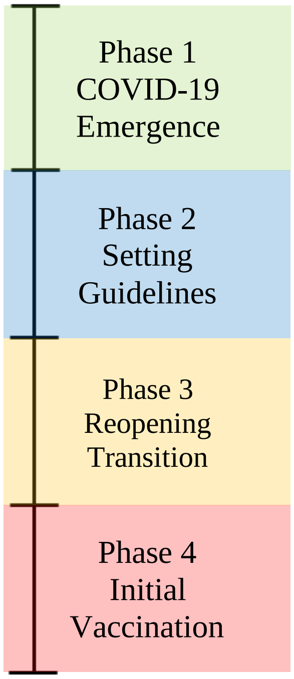

(c)

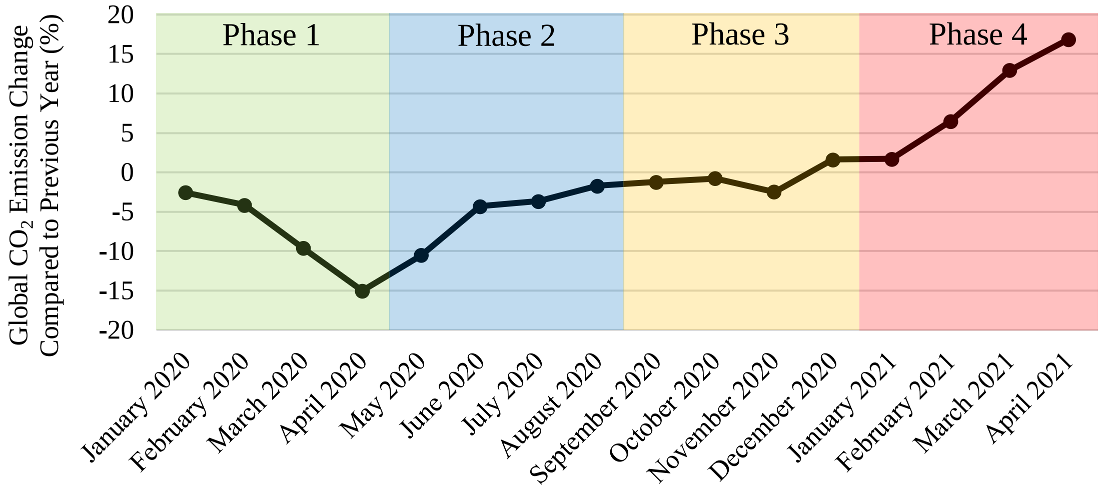

Supplement: Supplementary file 1 — Data Set S1 [file EFT2-10-0-s001.zip › supplementary_materials/figures_and_tables/figure_2.pdf]
