# Supplementary material for: Translated Emission Pathways (TEPs): Long‐Term Simulations of COVID‐19 CO2 Emissions and Thermosteric Sea Level Rise Projections
Source: Earths Future. 2022 Aug 24;10(8):e2021EF002453. doi: 10.1029/2021EF002453 (PMC9538853; doi:10.1029/2021EF002453)
Supplement: Supplementary file 1 — Data Set S1 [file EFT2-10-0-s001.zip › supplementary_materials/figures_and_tables/table_1.pdf]

TEP Designation	RCP Resemblance	Phase Selection	Emission Translation	Monthly Multiplier	Emission Order
TEP Mitigation	RCP 2.6	Phase 1	20 Years	x2	Reverse Order
TEP Shutdown	RCP 4.5	Phase 1 and 2	10 Years	x1	Original Order
TEP Reopening	RCP 6.0	Phase 3 and 4	10 Years	x2	Reverse Order
TEP Unregulated	RCP 8.5	Phase 4	20 Years	x4	Reverse Order
