# Supplementary figures and images for: Translated Emission Pathways (TEPs): Long‐Term Simulations of COVID‐19 CO2 Emissions and Thermosteric Sea Level Rise Projections
Source: Earths Future. 2022 Aug 24;10(8):e2021EF002453. doi: 10.1029/2021EF002453 (PMC9538853; doi:10.1029/2021EF002453)

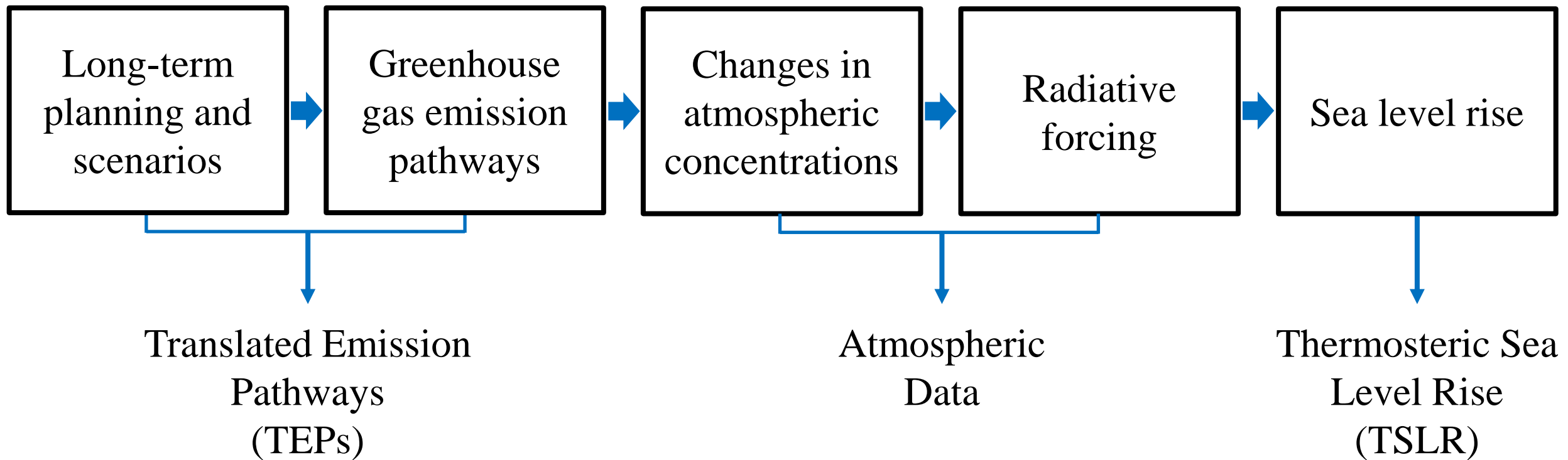

Supplement: Supplementary file 1 — Data Set S1 [file EFT2-10-0-s001.zip › supplementary_materials/figures_and_tables/figure_1.pdf]

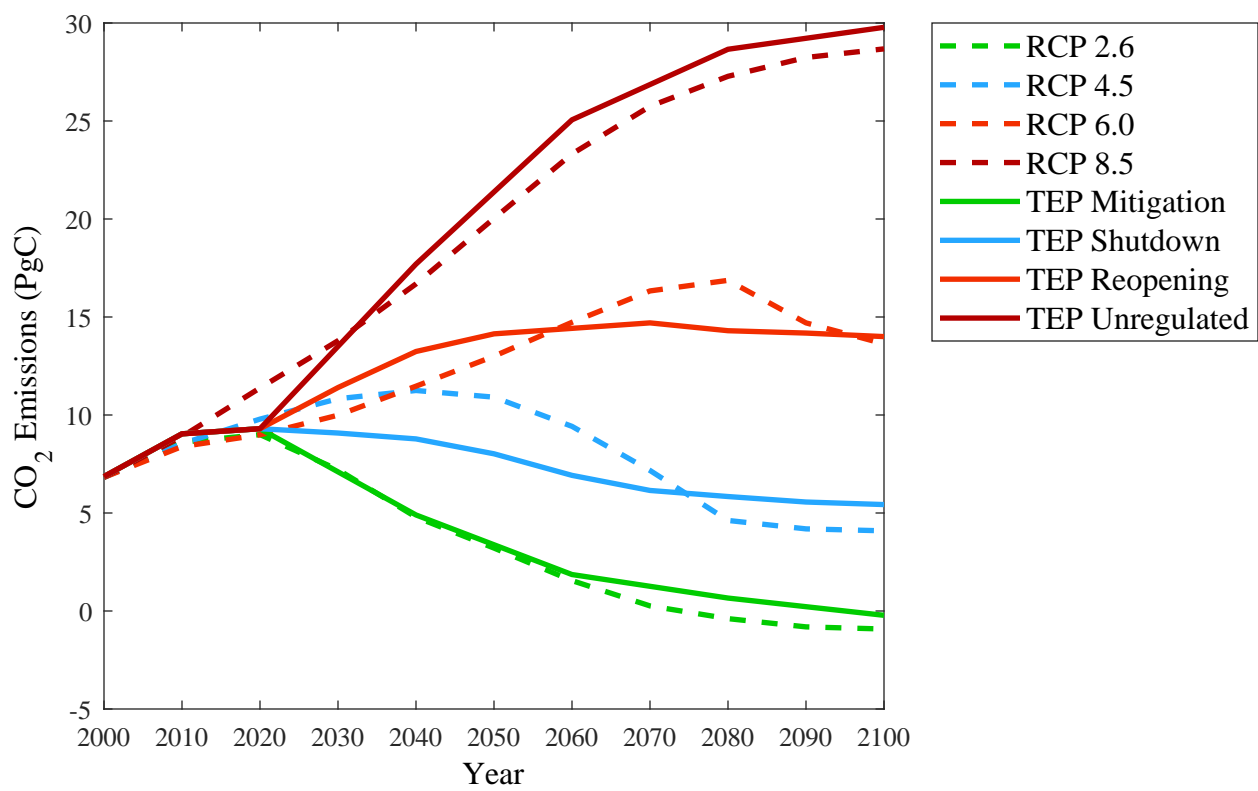

Supplement: Supplementary file 1 — Data Set S1 [file EFT2-10-0-s001.zip › supplementary_materials/figures_and_tables/figure_3.pdf]

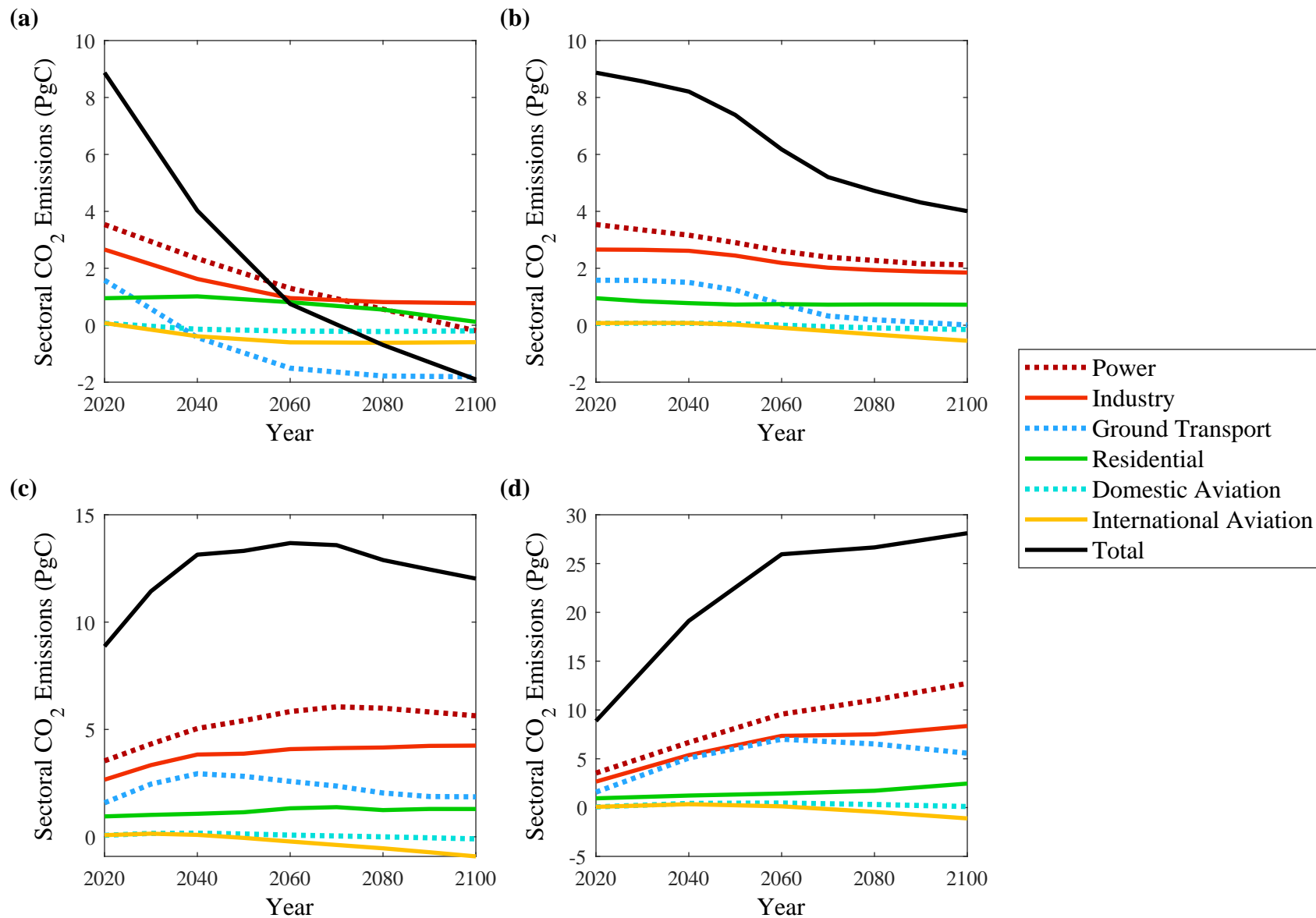

Supplement: Supplementary file 1 — Data Set S1 [file EFT2-10-0-s001.zip › supplementary_materials/figures_and_tables/figure_4.pdf]

(a)

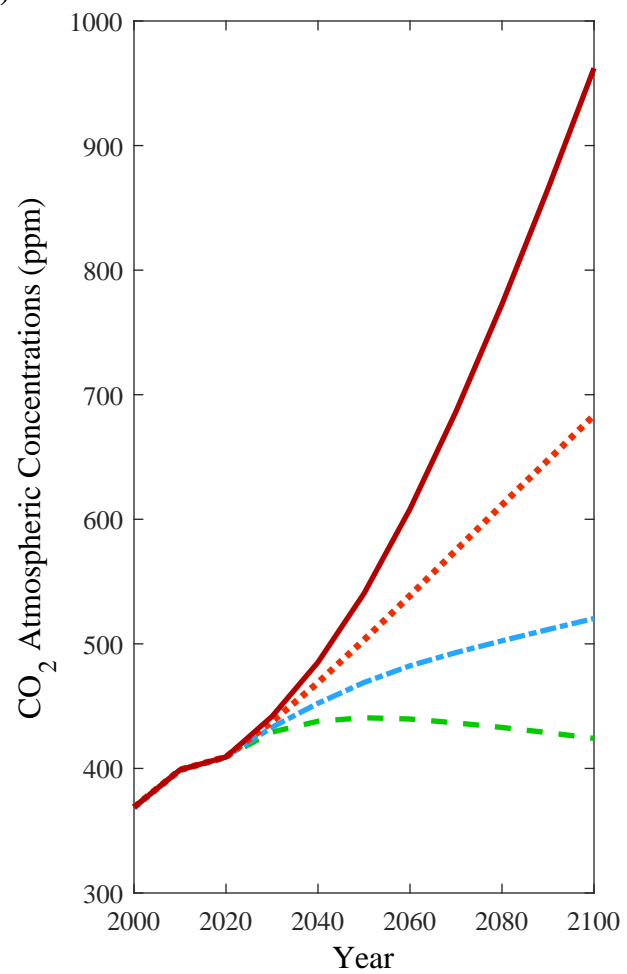

(b)

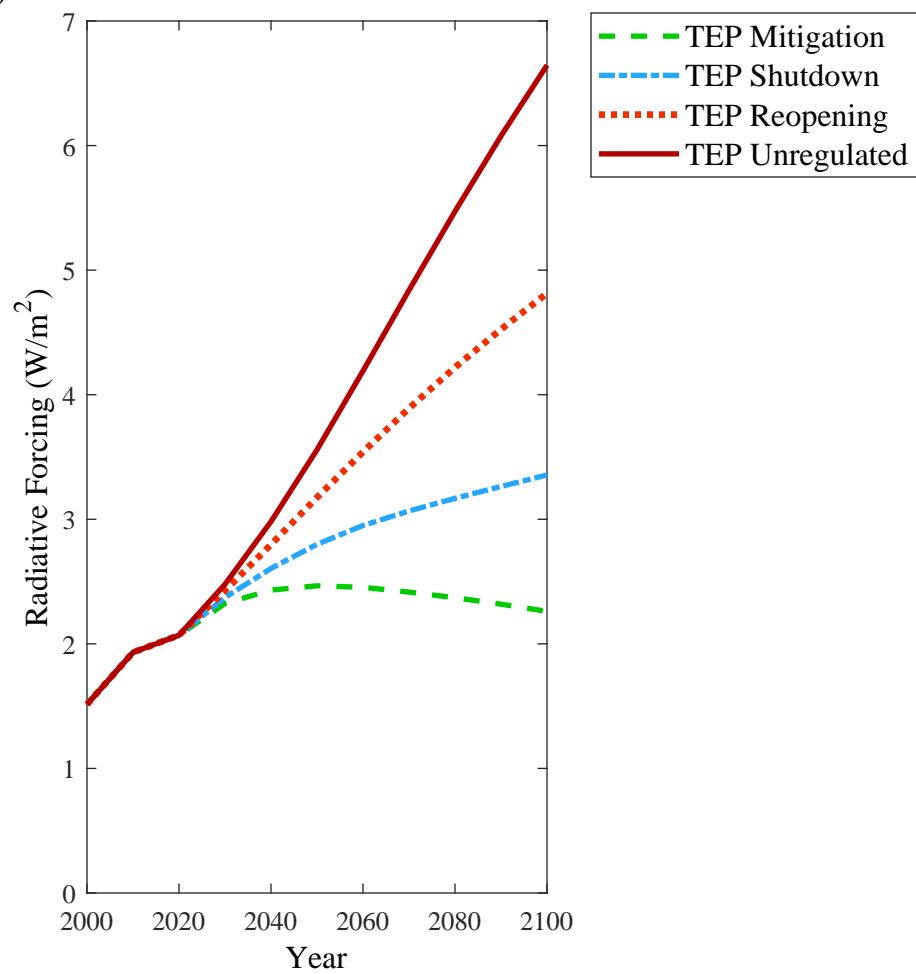

Supplement: Supplementary file 1 — Data Set S1 [file EFT2-10-0-s001.zip › supplementary_materials/figures_and_tables/figure_5.pdf]

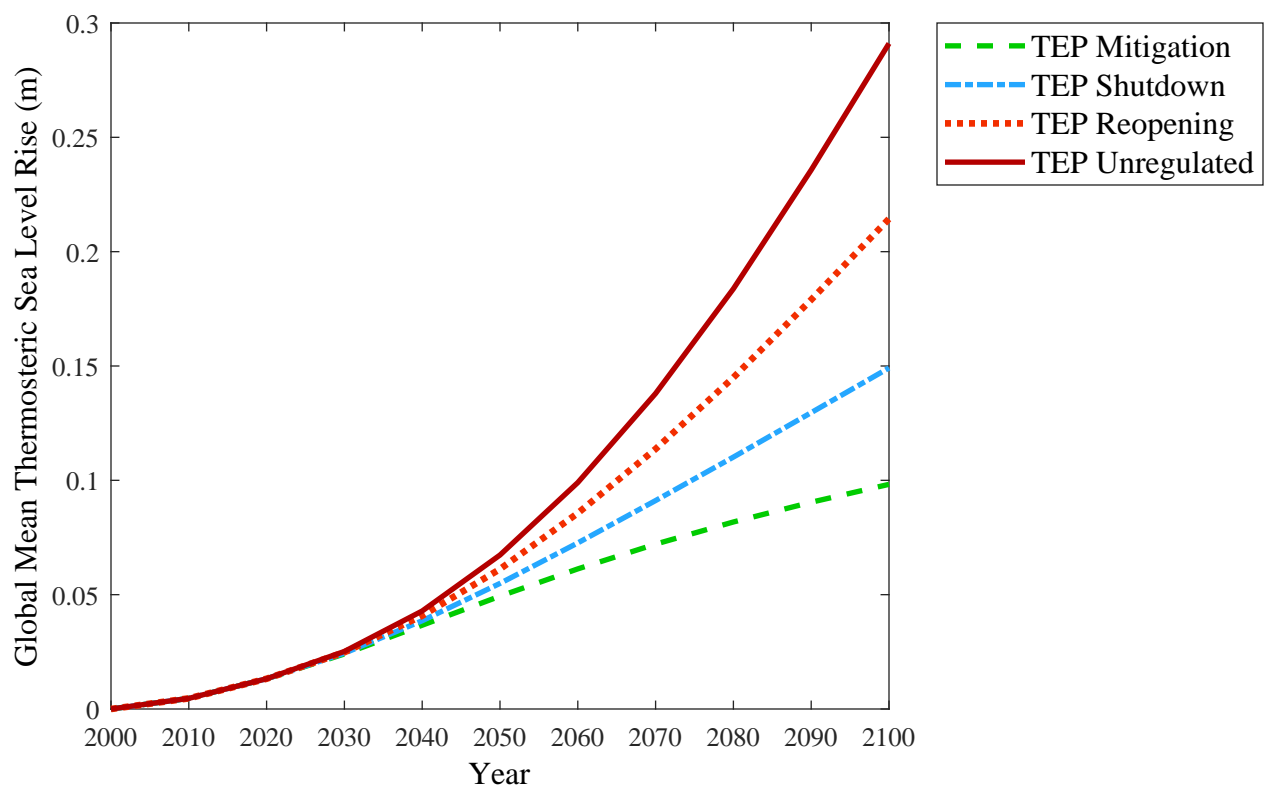

Supplement: Supplementary file 1 — Data Set S1 [file EFT2-10-0-s001.zip › supplementary_materials/figures_and_tables/figure_6.pdf]
